# Fish oil ameliorates ethanol-induced gastric injury in rat by modulating gene related to apoptosis

**DOI:** 10.1038/s41598-024-56647-5

**Published:** 2024-03-14

**Authors:** Nikoo Parham, Kaveh Rahimi, Zohreh Ghotbeddin, Mohammad Reza Tabandeh

**Affiliations:** 1 Department of Basic Sciences, Faculty of Veterinary Medicine, Shahid Chamran University of Ahvaz, Ahvaz, Iran; 2 Department of Basic Sciences, Division of Biochemistry and Molecular Biology, Faculty of Veterinary Medicine, Shahid Chamran University of Ahvaz, Ahvaz, Iran; 3 Stem Cells and Transgenic Technology Research Center, Shahid Chamran University of Ahvaz, Ahvaz, Iran

**Keywords:** Fish oil, Ethanol, Gastric injury, Apoptosis, Biochemistry, Cell biology, Physiology, Gastroenterology

## Abstract

Gastric ulcers are a type of digestive disease that can severely affect a person's quality of life. Our study aimed to investigate the effects of fish oil on ethanol-induced gastric ulcers in rats, with the purpose of providing more comprehensive information on the topic. The study looked at various factors such as gastric ulcer index, and nitric oxide (NO) levels in stomach tissue. To investigate apoptosis, the mRNA levels of Bax, Bcl-2, and Caspase 3 were analyzed. The results showed that fish oil can reduce gastric acidity and the gastric ulcer index in cases of ethanol-induced gastric ulcers. It was found that fish oil can increase NO levels and improve the anti-apoptotic system by increasing the expression of Bcl-2 while decreasing the expression of Bax and Caspase 3. In general, the study demonstrates that fish oil can protect the stomach from ethanol-induced damage by reducing the apoptosis pathway via nitric oxide.

## Introduction

Fish oil is rich in long-chain n-3 polyunsaturated fatty acids (PUFAs) like eicosapentaenoic acid (EPA) and docosahexaenoic acid (DHA)^1^. PUFAs serve as the building blocks for eicosanoids, a group of signaling molecules that regulate various physiological processes. Among PUFAs, arachidonic acid (20:4, n-6) is the primary precursor for eicosanoid synthesis. Enzymatic transformation of arachidonic acid can lead to the production of prostaglandins or leukotrienes with two or four double bonds, respectively, which are known to have pro-inflammatory effects. On the other hand, eicosanoids synthesized from n-3 fatty acids contain three and five double bonds, respectively, and are less biologically active than those derived from n-6 fatty acids^2^. The consumption of fish oil supplements can reduce the production of inflammatory cytokines like interleukin-1 (IL-1) and tumor necrosis factor (TNF)^3^. Leukocyte-produced reactive oxygen species (ROS) can be reduced by n-3 fatty acids^4^. EPA and DHA are essential for the production of resolvins, which aid in the resolution of inflammation^5^. Additionally, long-chain n-3 fatty acids have anti-apoptotic properties^6^.

Fish oil derived from Scomberoides commersonianus reduced the injury severity in the gastric ulcer model induced by cold stress (CRS), aspirin, alcohol, and pyloric ligation. The fish oil has increased the activity of antioxidant enzymes (glutathione peroxidase and catalase) and decreased lipid peroxidation in the gastric mucosa of mice under CRS^7^. The various chemical drugs used to treat peptic ulcers. However, no life-threatening effects have been reported with antiulcer medications. However, many side effects, including constipation, abdominal pain, indigestion, bloating, diarrhea, nausea, and vomiting have been reported^8^. Studying compounds of animal and plant origin with fewer side effects is necessary for the treatment of gastrointestinal ulcers^9^.

Stomach damage is a common disorder in humans and animals. The breakdown of the gastric mucosal barrier is the leading cause of gastritis and gastric ulcers. Stomach foreign bodies and non-observance of diet are common causes of acute gastritis in humans and small animals including, dogs and cats^10^. One of the most widely used methods for inducing gastric damage in laboratory animals is ethanol, which is similar to acute damage in the stomach^11^. The ethanol digests the mucous layer and exposes the mucosa to the proteolysis activities of pepsin^12^.

Nitric oxide (NO) is a molecule with diverse functions in biology, having both positive and negative effects. Its biological effects are complex due to its interactions with other molecules like reactive oxygen species (ROS), metal ions, and proteins. The effects of NO can be affected by multiple factors such as the type of cell and the dosage^13^. NO can help to protect the gastric ulcer mucosal lesions. The effects of NO may be realized by its anti-angiogenic, anti-inflammatory and anti-apoptotic effects^14^.

The cell response to an apoptotic stimulus depends on the balance between anti-apoptotic proteins (Bcl-2 and Bcl-xl) and pro-apoptotic proteins (Bax, Bad, and Bac)^15^.

The NO has anti-apoptotic effects, which it does through various mechanisms, such as inactivation of many caspases, including caspase 3, as well as upregulation of Bcl-2 and Bcl-XL^16^. So far, there is no known information about the relationship between nitric oxide and apoptosis in the gastric ulcer model induced by ethanol. Our study intends to examine the effect of fish oil on nitric oxide levels and the changes in genes related to the apoptosis pathway in rats with ethanol-induced gastric ulcers.

## Material and methods

### Animals

Wistar rats (240–260 g) were obtained. The animals were kept in plexiglass cages with a 12-h light and 12-h dark cycle at 23 ± 2 °C. The rats were fed rat chow and provided water ad libitum. In the following reported experiments on live vertebrates and methods used all relevant guidelines and regulations, particularly ARRIVE guidelines27, have been fully considered and noticed^17^. Furthermore, the protocol of the current study was approved by the Research Ethics Committee of the Faculty of Veterinary Medicine, Shahid Chamran University of Ahvaz, Ahvaz, Iran (EE/1401.2.24.226074/scu.ac.ir).

### Experimental design

**Figure 1:**
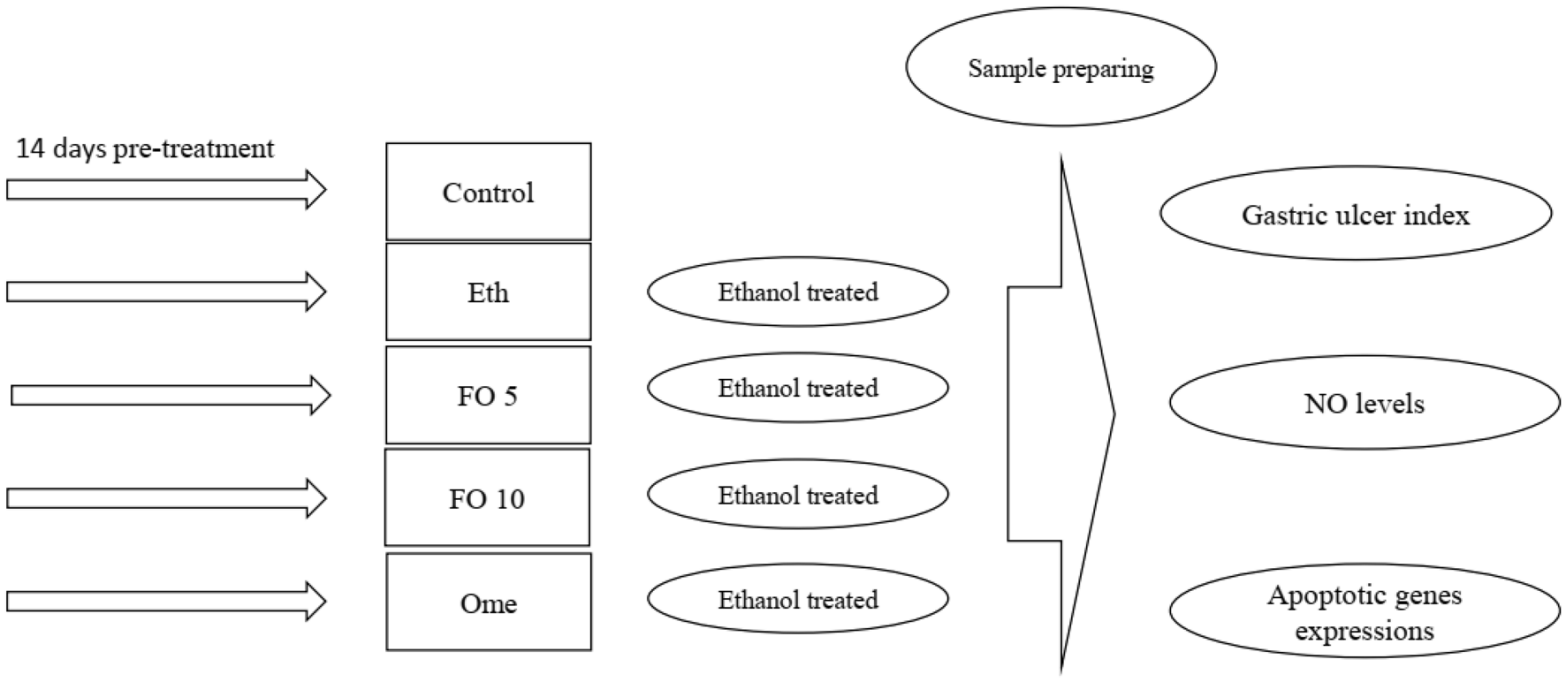
Graphical abstract.

The rats in this experiment were divided into five groups, each with six rats. In the treatment groups, fish oil was administered orally at either 5 or 10 ml/kg BW^18^ (Sigma-Aldrich CAS Number: 8002-50-4) for 14 days before the administration of ethanol^19^. The positive control group received omeprazole at 20 mg/kg^19^, while the ethanol group was given sunflower oil for 14 days before the administration of ethanol. The control group received only sunflower oil. On the 14th day, 90 min after each ethanol administration, the rats were sacrificed and their gastric tissues were harvested as shown in Fig. 1.

### Macroscopic assessment

The stomachs were removed from the greater curvature and rinsed with normal saline. The ulcer size was measured with a vernier caliper and the percentage of inhibition was calculated. Ulcer inhibition% = (GUI in the model − GUI in test) × 100/GUI in model.

### Measurement NO levels

The nitric oxide levels in the gastric mucosa were measured following the NO kit instructions (NO, Kiazist, Hamedan, Iran cat#KNO96). Thus, after homogenizing the tissue, the amount of protein was calculated according to the Bradford method. Then, NO was measured according to the instructions of the manufacturer of the kit.

### Real-time PCR

Real-time PCR was performed for Bax, Bcl2, and caspase-3. The mRNA was prepared using Total RNA Extraction Kit (Parstous, Iran). The cDNA from total RNA with a cDNA synthesis kit (Parstous, Iran) was synthesized. All primers’ sequences for qPCR listed in Table 1. The quantitative real-time PCR was performed using real-time PCR Master Mix (SYBR® Green-Parstous, Iran). The data was calculated through the 2^−△△CT^ method with GAPDH as an endogenous reference.

**Table 1:** Primer sequences.

Gene name	Sequence	Length	Accession no.
GAPDH-rat-F	AGTTCAACGGCACAGTCAAG	119	XM_017593963.1
GAPDH-rat-R	TACTCAGCACCAGCATCACC		
bax-rat-F	TGCTACAGGGTTTCATCCAG	144	NM_017059.2
bax-rat-R	TGTTGTTGTCCAGTTCATCG		
bcl2-rat-F	ATCGCTCTGTGGATGACTGAGTAC	135	L14680.1
bcl2-rat-R	AGAGACAGCCAGGAGAAATCAAAC		
caspase 3-rat-F	CTATCCATGGAAGCAAGTCGATG	136	NM_012922.2
caspase 3-rat-R	TTGCGAGCTGACATTCCAGT		

### Statistical analysis

We analyzed the data using SPSS software version 16. First, we checked the data for normal distribution and homogeneity of variances using the Kolmogorov–Smirnov test in SPSS. Since the data met the normality test, we performed a one-way analysis of variance (ANOVA) to compare the groups. We used Tukey test for post-hoc analyses. We analyzed ulcer index data using the Kruskal–Wallis test. *p < 0.05, **p < 0.01 and ***p < 0.001 were considered statistically significant.

## Results

### Effect of fish oil pre-treatment on macroscopic inspection of gastric mucosa: Gastric Ulcer Index (GUI)

**Figure 2:**
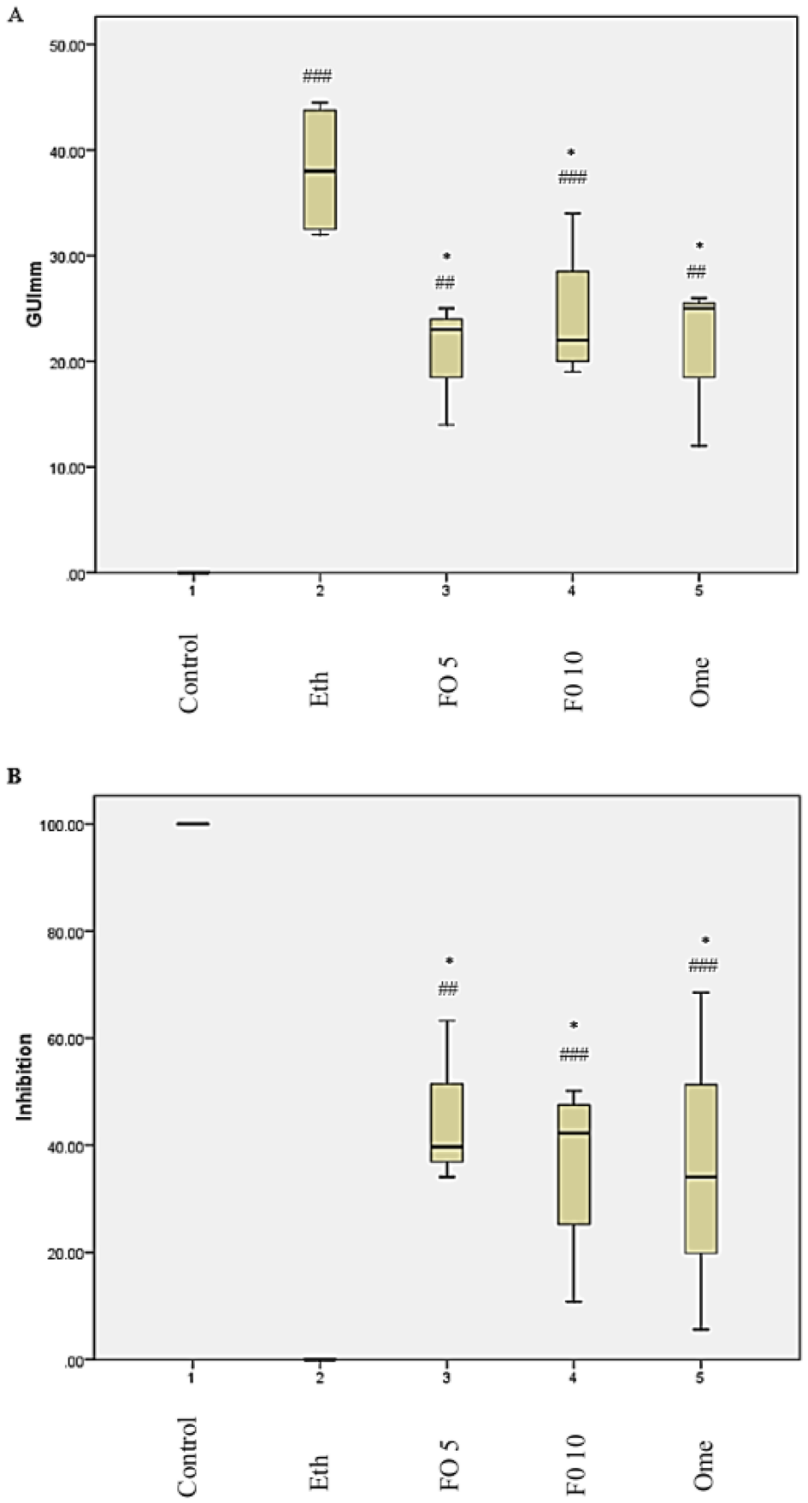
Effect of fish oil on (**A**) Gastric ulcer index (**B**) Ulcer inhibition in ethanol-induced gastric mucosa injury. (1) Control, (2) Ethanol (Eth), (3) Fish oil 5 ml/kg, (4) Fish oil 10 ml/kg, and (5) Omeprazole 20 mg/kg. ^##^p < 0.05 and ^###^p < 0.01 when compared with the control group. *p < 0.05 when compared with the Ethanol (Eth) group.

**Figure 3:**
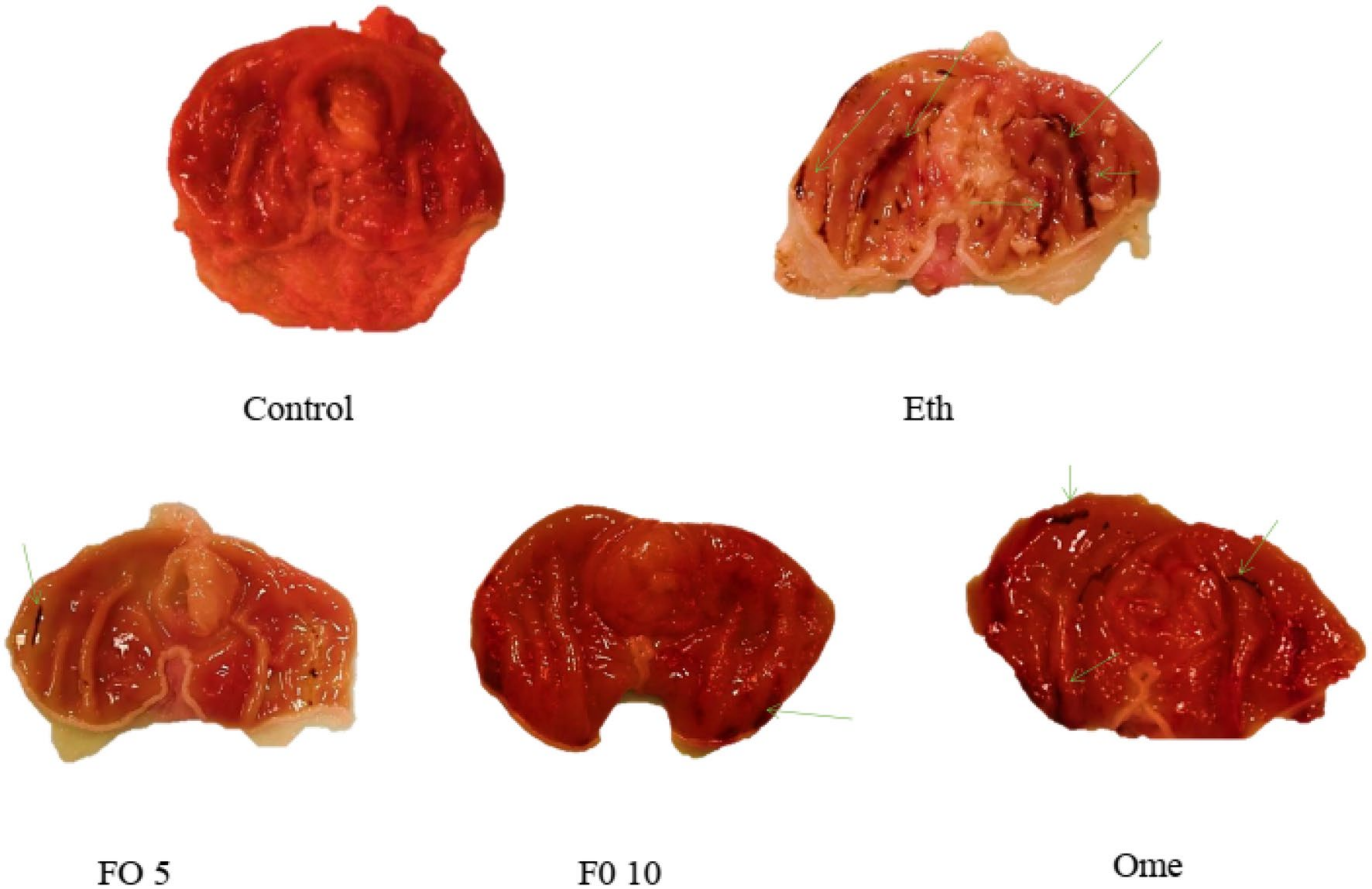
Gross macroscopic appearance of the gastric mucosa of Control, Ethanol (Eth), Fish oil (FO) 5 ml/kg, Fish oil (FO) 10 ml/kg, and Omeprazole (Ome) 20 mg/kg.

Based on the findings of this study, it was discovered that the average wound size (measured in mm^2^) was significantly greater in the ethanol, fish oil 5 ml/kg, fish oil 10 ml/kg, and omeprazole 20 mg/kg groups compared to the control group (p < 0.001, p < 0.01, p < 0.001, and p < 0.01). On the other hand, the average wound size in mm^2^ was significantly lower in the 5 ml/kg fish oil, 10 ml/kg fish oil, and 20 mg/kg omeprazole groups when compared to the ethanol group (p < 0.05) (Figs. 2A and 3).

In the study, it was found that the groups given ethanol, fish oil 5 ml/kg, fish oil 10 ml/kg, and omeprazole 20 mg/kg had a significantly lower percentage of ulcer inhibition compared to the control group (p < 0.001, p < 0.001, p < 0.01, p < 0.001). On the other hand, the groups given 5 ml/kg fish oil, 10 ml/kg fish oil, and 20 mg/kg omeprazole had a significantly higher percentage of ulcer inhibition compared to the ethanol group (p < 0/05) (Figs. 2B and 3).

### Effect of fish oil pre-treatment on gastric mucosal NO levels

**Figure 4:**
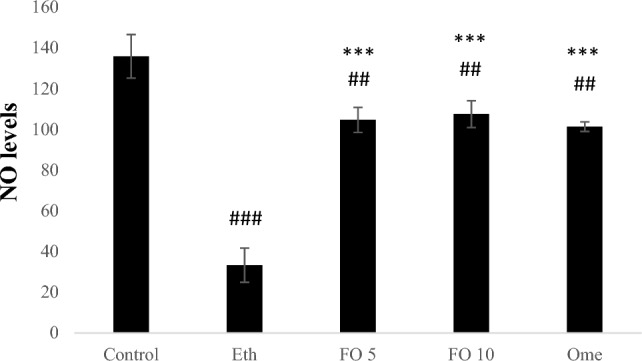
Effect of FO on NO levels. ^##^p < 0.05 and ^###^p < 0.01 when compared with the control group. ***p < 0.01 when compared with the Ethanol (Eth) group.

The groups administered with ethanol, fish oil at 5 ml/kg, fish oil at 10 ml/kg, and omeprazole at 20 mg/kg demonstrated significantly lower NO levels compared to the control group (with p-values of less than 0.001, 0.01, 0.01, and 0.01). On the other hand, NO levels were significantly higher in the 5 ml/kg fish oil, 10 ml/kg fish oil, and 20 mg/kg omeprazole groups compared to the ethanol group (with p-values of less than 0.001, less than 0.001, and less than 0.001) (Fig. 4).

### Effect of fish oil pre-treatment on gastric mucosal apoptotic genes expressions

**Figure 5:**
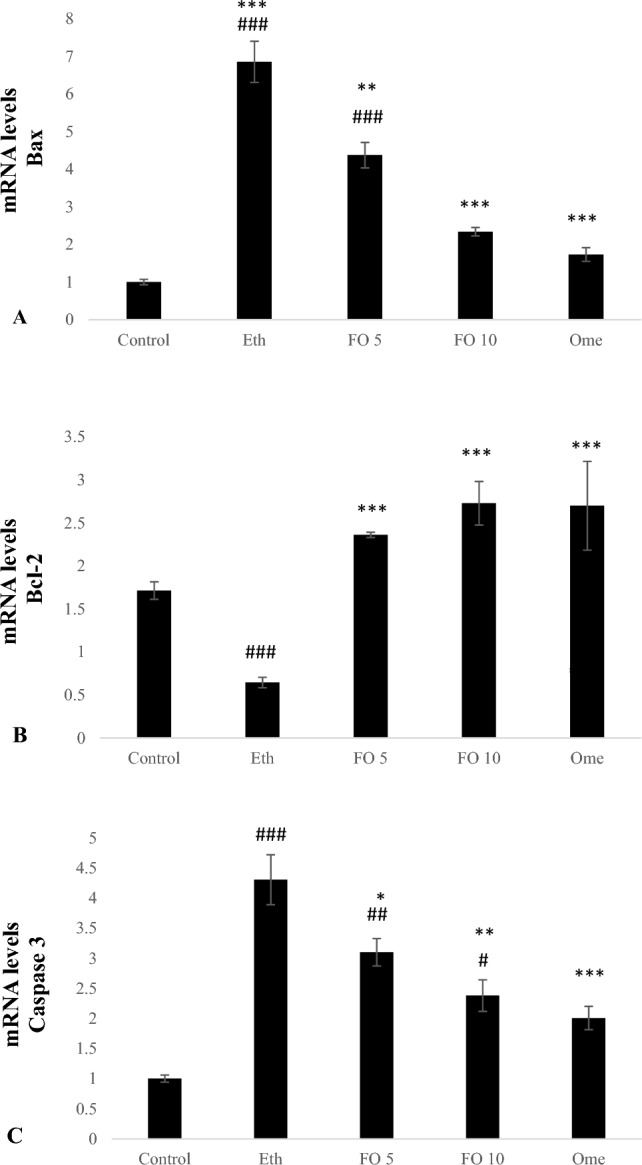
Effect of FO on (**A**) Bax mRNA levels (**B**) Bcl-2 mRNA levels (**C**) Caspase 3 mRNA levels. ^#^p < 0.05, ^##^p < 0.01 and ^###^p < 0.001 when compared with the control group. *p < 0.05, **p < 0.01 and ***p < 0.001 when compared with the Ethanol (Eth) group.

The groups treated with ethanol and fish oil 5 ml/kg showed a significant increase in the mRNA expression of Bax compared to the control group (p < 0.001 and p < 0.001). The mRNA expression level of Bax in the groups treated with fish oil 10 ml/kg and omeprazole 20 mg/kg did not differ significantly from the control group. However, the groups treated with 5 ml/kg fish oil, 10 ml/kg fish oil, and 20 mg/kg omeprazole showed a significant decrease in the mRNA expression of Bax compared to the group treated with ethanol (p < 0.01, p < 0.001, and p < 0.001) (Fig. 5A).

The ethanol group showed significantly lower Bcl-2 mRNA expression than the control group (p < 0.001). The 5 and 10 ml/kg fish oil and 20 mg/kg omeprazole groups did not exhibit any significant differences in the level of Bcl-2 mRNA expression compared to the control group. However, the mRNA expression of Bcl-2 in the 5 ml/kg fish oil, 10 ml/kg fish oil, and 20 mg/kg omeprazole groups was significantly higher than that of the ethanol group (p < 0.01, p < 0.001, and p < 0.001) as shown in Fig. 5B.

The amount of Caspase 3 mRNA expression was found to be significantly higher in the groups treated with ethanol and fish oil at doses of 5 and 10 ml/kg, compared to the control group (p < 0.001, p < 0.01 and p < 0.05). However, in the group treated with omeprazole at a dose of 20 mg/kg, there was no significant difference in the level of Caspase 3 mRNA expression when compared to the control group. Interestingly, the groups treated with 5 and 10 ml/kg of fish oil and 20 mg/kg of omeprazole had significantly lower levels of Caspase 3 mRNA expression when compared to the ethanol group (p < 0.05, p < 0.01 and p < 0.001) (Fig. 5C).

## Discussion

In recent decades, numerous epidemiological studies have investigated the abundant health benefits of omega-3 PUFAs. Numerous clinical studies have demonstrated the advantages of fish oil^20^. Research has found that fish oil can help prevent damage to the stomach lining caused by stress, certain drugs, and harmful substances. When given at a dosage of 5 or 10 ml/kg of body weight, fish oil has been demonstrated to provide substantial protection in various experimental models. It can reduce damage caused by ulcers from pyloric ligation, indomethacin, aspirin, reserpine, or hypothermic restraint. Additionally, fish oil has been found to have a significant inhibitory effect on gastric mucosal lesions caused by different necrotizing agents^7,21^.

The ethanol-stimulated gastric ulcer model has been widely used to investigate the gastroprotective effect of various drugs and natural products^19,22–24^. In the current study, we observed that the damage caused by ethanol was severe in the group treated with ethanol.

NO mediates critical physiological functions in the body, including the digestive system. The NO plays a role in the digestive system in regulating mucosal blood flow and maintaining the integrity of the mucosa. The NO also stimulates mucus secretion in gastric mucosal cells^25^. The NO plays a vital role in the defense of the gastrointestinal tract in pathological conditions^26^. The studies have shown that NO has protects against NSAID-induced gastric ulcers^27^. Ethanol induces hypersecretion of gastric acid, proinflammatory cytokines, and ROS. These factors function together to induce apoptosis and reduce the levels of NO^28–30^. Consistent with previous studies in our study, ethanol decreased NO content. The process by which different doses of ethanol reduces nitric oxide levels in the gastric tissue has not been fully understood. However, the reduction of NO is likely not caused by ethanol's effect on NO biosynthesis, but rather by its impact on the nitric oxide derived from the gastric tissue, ultimately leading to a decrease in NO^31^. However, like to omeprazole, fish oil 5 and 10 ml/kg significantly increased NO levels compared to the control group.

Evidence has shown that mitochondrial damage during apoptosis is closely related to Bax and Caspase-3 genes, which disrupt the integrity of gastric mucosa after ethanol administration^32–34^. The oxidative stress caused by ethanol causes the accumulation of free radicals derived from oxygen in the wound areas, the resistance of the antioxidant defense system decreases, and the intrinsic pathway of apoptosis is activated. In the intrinsic pathway of apoptosis, the activation of Bax causes the release of other pro-apoptotic factors and the formation of apoptosomes stimulated, and finally, caspase-3 is activated^35,36^. The current study showed that fish oil 5 and 10 ml/kg pretreatment decreased the expression of Bax and Caspase-3 in ethanol-induced rats. Also, fish oil 5 and 10 ml/kg pretreatment increases the expression of Bcl-2 in ethanol-induced rats. Similar effects observed for omeprazole.

The beneficial role of fish oil in gastric ulcers has been shown by inhibiting the invading mucosal factors and strengthening the defensive mucosal factors^7,37^. Fish oil increase the activity of the l-arginine-NO pathway and NO synthase (NOS) expression^38^. Fish oil supplementation increases NO and reduce inflammation, and, thus that can improve antioxidant capacity^39^. NO has a crucial role in the mechanism of gastric mucosal protection and injury induced by ethanol^40^. Its apoptotic and anti-apoptotic response depends on the cell type. NO activates apoptosis in many types of cells, including pancreatic islets, macrophages, thymocytes, and neurons. However, NO shows anti-apoptotic properties in gastric mucosa. Several anti-apoptotic mechanisms have demonstrated for NO^13^. Once apoptosis is triggered, proenzymes are cleaved into subunits that rearrange to form active cysteine proteases, activating caspases. Initiator caspases enhance the apoptotic signal by activating other executor caspases. In this work, the substrate used primarily measures the activity of executor caspases, such as caspase 3. A significant aspect of apoptosis is the condensation of chromatin and the specific cleavage of double-stranded DNA. This process involves the caspase-dependent removal of an inhibitory subunit from a DNAase, which is then transferred to the nucleus^41^. The NO inhibits caspase activation in both death-ligand-dependent and apoptosis-ligand-dependent ways. It has stated that the regulation of caspase activation by NO causes changes in the downstream genes, namely Bcl-2 and Bax. The NO prevented the reduction of Bcl-2 protein expression and its mRNA levels and also prevented the increase of Bax^13^.

## Conclusion

Our study showed that the administration of fish oil has a protective role against gastric damage caused by oral ethanol administration. The observed effects are probably due to the change in NO levels in the gastric tissue. As a result, the expression of genes related to apoptosis decreases, and the expression of anti-apoptotic genes increases.

## Data Availability

The datasets used and/or analyzed during the current study available from the corresponding author on reasonable request.
